# Characterization of the Hemolytic Activity of Mastoparan Family Peptides from Wasp Venoms

**DOI:** 10.3390/toxins15100591

**Published:** 2023-09-28

**Authors:** Xiangdong Ye, Huajun Zhang, Xudong Luo, Fengyin Huang, Fang Sun, Liangbin Zhou, Chenhu Qin, Li Ding, Haimei Zhou, Xin Liu, Zongyun Chen

**Affiliations:** 1 Department of Biochemistry and Molecular Biology, Institute of Basic Medical Sciences, College of Basic Medicine, Hubei University of Medicine, Shiyan 442000, China; yexiangdong@hbmu.edu.cn (X.Y.); z1269658612@163.com (H.Z.); luoxudong@hbmu.edu.cn (X.L.); 2016202040055@whu.edu.cn (F.S.); 2016202040025@whu.edu.cn (C.Q.); zhm030528@126.com (H.Z.); liuxin00113@126.com (X.L.); 2 Hubei Key Laboratory of Wudang Local Chinese Medicine Research, Hubei University of Medicine, Shiyan 442000, China; 3 Department of Public Studies, Changde Vocational Technical College, Changde 415000, China; phoenixhuang1996@foxmail.com; 4 Department of Orthopedics and Traumatology, Prince Wales Hospital & Faculty of Medicine, The Chinese University of Hong Kong, Hong Kong 999077, China; jacobchou@163.com; 5 Department of Clinical Laboratory, Dongfeng Hospital, Hubei University of Medicine, Shiyan 442000, China; dl2168@163.com

**Keywords:** wasp venom, mastoparan family peptide, hemolytic activity, antimicrobial activity

## Abstract

Biologically active peptides have attracted increasing attention in research on the development of new drugs. Mastoparans, a group of wasp venom linear cationic α-helical peptides, have a variety of biological effects, including mast cell degranulation, activation of protein G, and antimicrobial and anticancer activities. However, the potential hemolytic activity of cationic α-helical peptides greatly limits the clinical applications of mastoparans. Here, we systematically and comprehensively studied the hemolytic activity of mastoparans based on our wasp venom mastoparan family peptide library. The results showed that among 55 mastoparans, 18 had strong hemolytic activity (EC_50_ ≤ 100 μM), 14 had modest hemolytic activity (100 μM < EC_50_ ≤ 400 μM) and 23 had little hemolytic activity (EC_50_ > 400 μM), suggesting functional variation in the molecular diversity of mastoparan family peptides from wasp venom. Based on these data, structure–function relationships were further explored, and, hydrophobicity, but not net charge and amphiphilicity, was found to play a critical role in the hemolytic activity of mastoparans. Combining the reported antimicrobial activity with the present hemolytic activity data, we found that four mastoparan peptides, Parapolybia-MP, Mastoparan-like peptide 12b, Dominulin A and Dominulin B, have promise for applications because of their high antimicrobial activity (MIC ≤ 10 μM) and low hemolytic activity (EC_50_ ≥ 400 μM). Our research not only identified new leads for the antimicrobial application of mastoparans but also provided a large chemical space to support the molecular design and optimization of mastoparan family peptides with low hemolytic activity regardless of net charge or amphiphilicity.

## 1. Introduction

Recently, wasp stings have become an increasingly serious public health problem because of the high incidence and mortality rates [1,2]. Hemolysis is one of the most common and prominent clinical symptoms of wasp stings, and the hemolysis incidence in severe patients (generally >30 skin sting lesions) is up to 83.5% [3]. The major complications in severe patients include acute kidney injury (AKI), liver damage and acute respiratory distress syndrome (ARDS), which are related to hemolysis; for example, hemolysis and rhabdomyolysis induced by massive wasp stings lead to the precipitation of haemoglobin and myoglobin casts in renal tubules, which cause AKI [4,5]. Multiple studies have found that wasp venom peptides can cause hemolysis by direct or indirect mechanisms: (1) Some wasp venom peptides can interact with the phospholipid bilayer of the cell membrane, leading to osmotic pressure changes in red blood cells and, thus, hemolysis [6,7]. (2) some wasp venom peptides can also activate phospholipase A on the erythrocyte membrane, mediating the hydrolysis of lecithin and resulting in hemolysis [8]. (3) Wasp venom peptides can prolong prothrombin time and reduce the activity of coagulation factors VIII and IX, leading to coagulation dysfunction [9,10].

Wasp venoms are rich in a variety of polycationic peptides and mastoparan family peptides account for approximately 50–60% of the dry weight of wasp venom [11]. Several studies have identified many mastoparans in different wasp venoms, and most mastoparans have abilities to induce hemolysis [12,13,14,15]; however, the relationship between the diversity of the molecular compositions and the hemolytic activity of wasp mastoparan family peptides remains unclear.

Mastoparan family peptides are multifunctional peptides that activate protein G and have antimicrobial, anticancer and hemolytic activities [14,15]. Mastoparan family peptides have been extensively studied for antimicrobial properties because of their unique cationic α-helix structure with amphipathicity [14]. It is reported that many mastoparans have antibacterial activity against both Gram-positive and -negative bacteria, and Mastoparan-C and Mastoparan-M, even have activity against drug-resistant bacteria [14,15]. These studies indicate that wasp mastoparan family peptides are an important antimicrobial peptide resource with good clinical application prospects. However, hemolytic activity is an important factor limiting their clinical development and application.

Here, based on our library of wasp mastoparan family peptides containing 55 mastoparans from 31 wasp species, as described in a previous study [16], we systematically and comprehensively investigated the hemolytic activity of 55 mastoparans towards human red blood cells (HRBCs) and rat red blood cells (RRBCs) and preliminarily explored the relationships between the molecular diversity, physical properties and hemolytic activity. By combining this information with the antibacterial activities of 34 reported mastoparans, we identified 4 AMP candidates with strong clinical application prospects. Our results provide a theoretical basis for the research and development of clinical drugs for hemolysis caused by wasp stings and provide basic guidance for the clinical treatment of wasp stings. Moreover, we identified four lead molecules, antimicrobial peptides with low hemolytic activity, to lay the foundation for the future use of antimicrobial peptides in drug development.

## 2. Results

### 2.1. Characterization of the Hemolytic Activity of Mastoparan Family Peptides from Wasp Venoms

**Figure 1:**
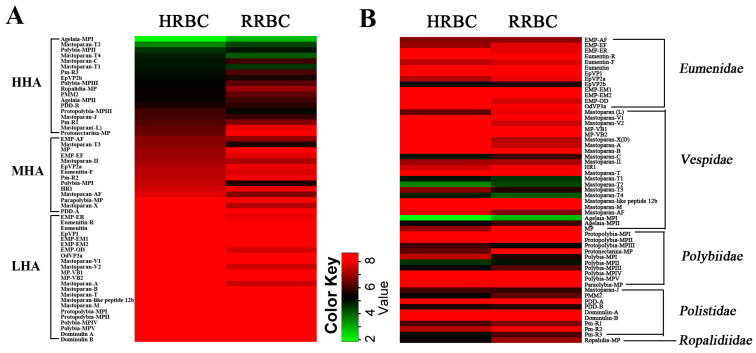
The heat map exhibition of the hemolytic activity of mastoparan peptides on human blood red cells and rat blood red cells. (**A**) Heat map exhibition of hemolytic activity of mastoparan peptides based on the value of EC_50_. (**B**) Heat map exhibition of hemolytic activity of mastoparan peptides based on wasp families.

**Figure 2:**
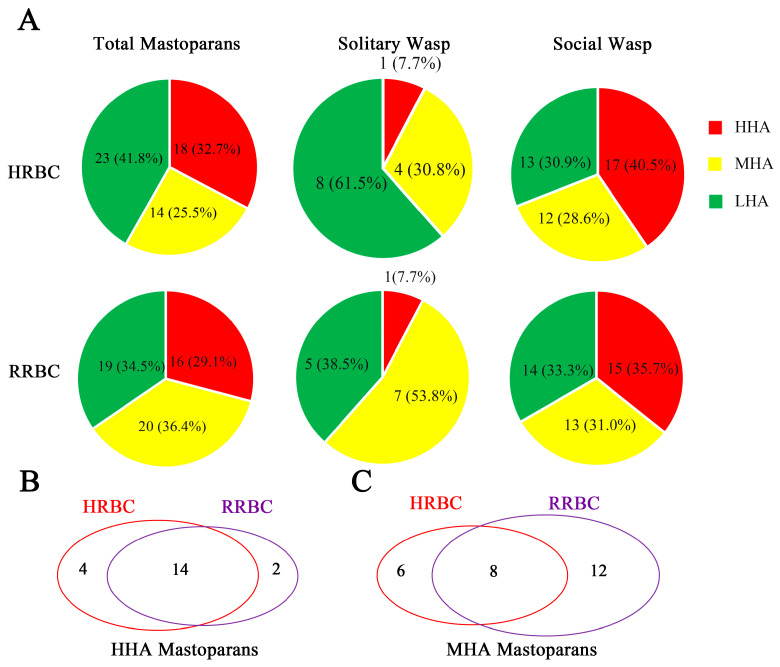
The hemolytic characteristic patterns of 55 mastoparans according to wasp lifestyles in HRBCs and RRBCs. (**A**) Pie chart analysis of the proportions of HHA, MHA and LHA among 55 mastoparans, divided into solitary wasps and social wasps. (**B**,**C**) Venn diagram analysis of the difference between HHA mastoparans (**B**) and MHA mastoparans (**C**) on HRBCs and RRBCs.

In a previous study, we established a wasp venom peptide library containing 55 mastoparan family peptides from 31 wasp species [16]. The molecular diversity of these 55 mastoparans and the results of previous studies suggest that the hemolytic activity of mastoparan peptides varies greatly. To investigate the hemolytic activity of whole mastoparan family peptides systematically and comprehensively, we examined the hemolytic activity of 55 mastoparans towards both human red blood cells (HRBCs) and rat red blood cells (RRBCs). The 55 mastoparans were divided into three groups depending on the hemolytic activity (EC_50_ values) towards HRBCs: a high-hemolytic-activity group (HHA, EC_50_ ≤ 100 μM), a modest-hemolytic-activity group (MHA, 100 μM < EC_50_ ≤ 400 μM or hemolysis rate ≥ 25% at 320 μg/mL), and a low-hemolytic-activity group (LHA, hemolysis rate < 25% at 320 μg/mL). As shown in Table 1 and Figure 1A, there were 18 mastoparans in HHA group, which accounted for 32.7% of 55 mastoparans, including Agelaia-MPI, Mastoparan-C, PMM2 and EpVP2b, which induced significant hemolysis of HRBCs in a dose-dependent manner with EC_50_ values of 3.7 ± 0.14 μM, 30.2 ± 1.3 μM, 42.6 ± 2.5 μM and 34.1 ± 3.5 μM, respectively (Figure S1A–D). A total of 14 mastoparans were in the modest-activity group, including Polybia-MPI, Mastoparan-II, MP and Eumenitin-F, with EC_50_ values of 176.6 ± 7.0 μM, 134.6 ± 1.2 μM, 123.6 ± 15.3 μM and 157.1 ± 2.6 μM, respectively (Figure S1E–H). Twenty-three mastoparans were in the low-activity group and had hemolytic activity too low to calculate the EC_50_ values, as shown in Table 1. We also investigated the hemolytic activity of mastoparans towards RRBCs. The results showed that the patterns of hemolytic activity of mastoparans towards RRBCs were similar to those towards HRBCs for HHA mastoparans (16 HHA mastoparans on RRBCs and 18 HHA mastoparans on HRBCs, 29.1% vs. 32.7%), and there were large differences in MHA mastoparans (20 vs. 14, 36.4% vs. 25.5%) between HRBCs and RRBCs (Figure 2A). The hemolytic curves of some mastoparans towards RRBCs are shown in Supplemental Figure S2. Nineteen mastoparans had little hemolytic activity (hemolysis rate lower than 25% at a dose of 320 μg/mL) towards both HRBCs and RRBCs, as shown in Table 1. For 36 hemolytic mastoparans, 24 mastoparans were more hemolytic towards HRBCs than RRBCs, such as mastoparan-C (30.2 ± 1.3 μM vs. 64.4 ± 10.7 μM), Mastoparan(-L) (82.9 ± 3.8 μM vs. 242.5 ± 2.6 μM) and Ropalidia-MP (42.5 ± 1.7 μM vs. 122.2 ± 4.3 μM), and 12 mastoparans were more hemolytic towards RRBCs than HRBCs, such as Polybia-MPI (51.4 ± 2.2 μM vs. 176.6 ± 7.0 μM) and Mastoparan-T3 (51.6 ± 2.1 μM vs. 112.1 ± 8.0 μM). These results showed that not all mastoparans could significantly induce the hemolysis of human red blood cells and that the hemolytic activity of mastoparans against HRBCs varies greatly.

Hemolysis curve of HRBCs induced by four HHA mastoparans: PMM2 (A), Mastoparan-C (B), Agelaia-MPI (C) and EpVP2b (D); and four MHA mastoparans: Polybia-MPI (E), Mastoparan-II (F), MP (G) and Eumenitin-F (H). Curves were generated by SigmaPlot 12.5 software and EC_50_ values were calculated according to the equation (four-parameter logistic curve): y = min + (max − min)/(1 + (x/EC_50_)^−Hillslope^).

Next, the sources of mastoparans with different hemolytic activities were investigated. First, wasps can be divided into two groups according to their habits: social wasps and solitary wasps. Our results show that the proportion of HHA mastoparans isolated from social wasp venom (40.4%, 17/42 in HRBCs and 35.7%, 15/42 in RRBCs) was much higher than that isolated from solitary wasp venom (7.7%, 1/13 in both HRBCs and RRBCs). Actually, the proportion patterns of hemolytic activity of total mastoparans are similar to those of 42 mastoparans from social wasps on both HRBCs and RRBCs. The main differences in mastoparan hemolytic activity between HRBCs and RRBCs lie in MHA mastoparans (4 vs. 7, 30.8% vs. 53.8%) and LHA mastoparans (8 vs. 5, 61.5% vs. 38.5%) from solitary wasps. Moreover, as shown in Figure 2B, the HHA mastoparans on HRBCs were consistent with those on RRBCs: 14 mastoparans had high hemolytic activity on both HRBCs and RRBCs. In contrast, the mastoparans with modest hemolytic activity differed greatly between HRBCs and RRBCs: only eight mastoparans had modest hemolytic activity on both HRBCs and RRBCs.

**Figure 3:**
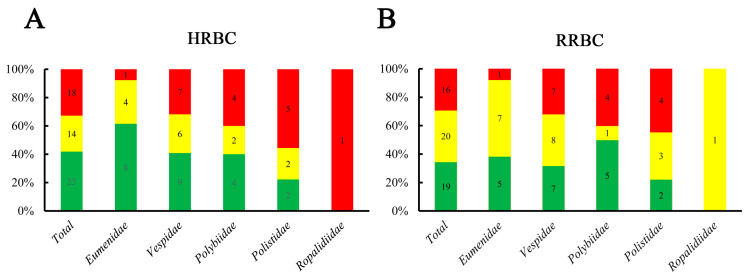
Characterization of the hemolytic activity of 55 mastoparans on wasp species. (**A**,**B**) Statistical analysis of the proportion and number of 55 mastoparans from different wasp families on HRBCs (**A**) and RRBCs (**B**). The red color means HHA mastoparans, yellow color means MHA mastoparans and green color means LHA mastoparans.

Considering that only *Eumenidae* wasps are solitary wasps and that the other four wasp families are social wasps, we next investigated the hemolytic activity of mastoparans isolated from *Polistidae, Polybiidae, Vespidae* and *Ropalidiidae*. The results showed that HHA mastoparans accounted for 55.6% (5/9) in *Polistidae*, which was much higher than the 32.7% (18/55) among 55 mastoparans, 40.0% (4/10) in *Polybiidae*, 31.8% (7/22) in *Vespidae* and 7.7% (1/13) in *Eumenidae* (Figure 2A). Interestingly, although there were only 7 HHA mastoparans in *Vespidae*, 5 mastoparans had the highest hemolytic activity among 55 mastoparans except for Polybia-MPII, namely, Agelaia-MPI (3.7 ± 0.14 μM), Mastoparan-T2 (11.8 ± 3.1 μM), Mastoparan-T4 (28.5 ± 2.3 μM), Mastoparan-C (30.2 ± 1.3 μM) and Mastoparan-T1 (30.8 ± 1.6 μM). Moreover, we also found that the LHA mastoparans accounted for 61.5% (8/13) of those derived from *Eumenidae*, which is much higher than the 41.8% (23/55) among all mastoparans, 40.9% in *Vespidae* (9/22), 40% in *Polybiidae* (4/10) and 22.2% (2/9) in *Polistidae*. These results showed that the ratio of HHA mastoparans in *Polistidae* was much higher than that in other wasp families, while the rate of HHA mastoparans from *Eumenidae* was the lowest. We also found that the hemolytic activity on HRBCs of some mastoparans derived from the same wasp family varied greatly, such as Mastoparan-C vs. Mastoparan-X(V) (30.2 ± 1.3 μM vs. 349.4 ± 4.9 μM), as did that of some mastoparans from the same wasp species, such as PDD-B and PDD-A (48.5 ± 3.4 μM vs. 353.5 ± 44.8 μM). These results indicated that the hemolytic activity of mastoparans varies greatly among species and families. We also found that the hemolytic activity characteristics of RRBCs and HRBCs in different wasp families were very similar (Figure 3A,B). These results suggest that the hemolytic mechanism of mastoparans on HRBCs and RRBCs may be similar, but the sensitivity of different red cells to mastoparans may vary. In our mastoparan family peptide library, there were four mastoparans (Eumenitin-F, Eumenitin-R, Eumenitin and EpVP1) without amidation modification on the C-terminus. Only Eumenitin-F had slight hemolytic activity on HRBCs, and the others had almost no hemolytic activity on either HRBCs or RRBCs. Our results suggest that amidation modification on the C-terminus of mastoparans may play an important role in the hemolytic activity of mastoparans.

### 2.2. Hydrophobicity Significantly Influences the Hemolytic Activity of Natural Mastoparan Peptides

**Figure 4:**
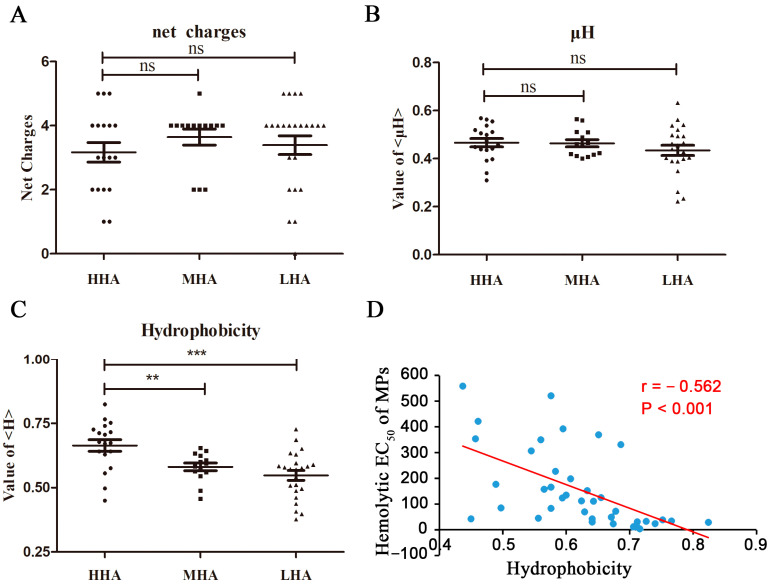
The hydrophobicity of peptides plays a critical role in the hemolytic activity of wasp mastoparan family peptides on HRBCs. (**A**–**C**) The effect of net charges (**A**), amphipathicity (μH, (**B**)) and hydrophobicity (**C**) of 55 mastoparans (18 HHA mastoparans, 14 MHA mastoparans and 23 LHA mastoparans) on hemolytic activity; ns: no significance, ** indicates that *p* value < 0.01 and *** indicates *p* value < 0.001. (**D**) Analysis of the relationship between the hemolytic EC_50_ values and hydrophobicity of 55 mastoparans.

The hemolytic activity of different mastoparans varies greatly, even those derived from the same wasp venom; for example, the hemolytic EC_50_ of Protopolybia-MPIII was 23.0 ± 1.2 μM, and Protopolybia-MPI had almost no hemolytic activity. We explored the roles of net charge, amphipathicity (μH) and hydrophobicity in the hemolytic activity of mastoparans, and the results showed that there was no significant difference in net charge and μH among HHA, MHA and LHA mastoparans, as shown in Figure 4A,B. As shown in Figure 4C, the hydrophobicity of HHA was significantly higher than that of MHA (*p* < 0.05) and LHA (*p* < 0.001), and the hemolytic EC_50_ of mastoparans was negatively correlated with its hydrophobicity (r = −0.562, *p* < 0.001) (Figure 4D); that is, the hemolytic activity of mastoparans was positively correlated with its hydrophobicity. It is suggested that hydrophobicity is a critical factor that affects the hemolytic activity of natural wasp mastoparan family peptides.

### 2.3. Structure–Functional Relationship Studies of Protopolybia-MPIII and Protopolybia-MPI Showed That Hydrophobicity Also Significantly Influences the Hemolytic Activity of Designed Mastoparan Peptides

**Figure 5:**
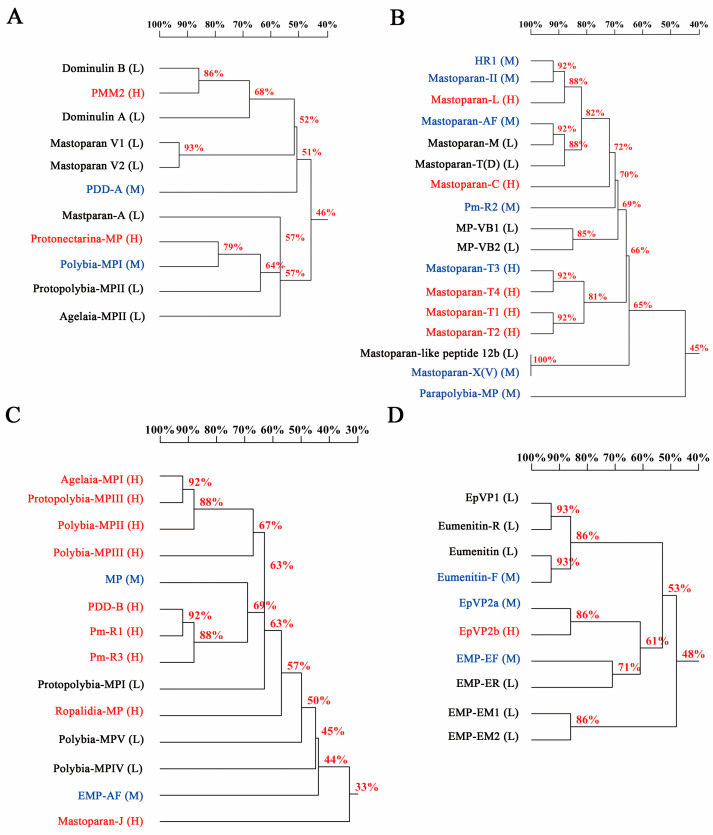
Structure–activity relationship analysis of 55 mastoparan family peptides on HRBCs. (**A**–**D**) Homology tree analysis of mastoparans in subfamily 1 (**A**), subfamily 2 (**B**), subfamily 3 (**C**) and subfamily 4 (**D**). Mastoparans are labelled in red (H means HHA, high hemolytic activity), blue (M means MHA, modest hemolytic activity) and black (L means LHA, low hemolytic activity).

**Figure 6:**
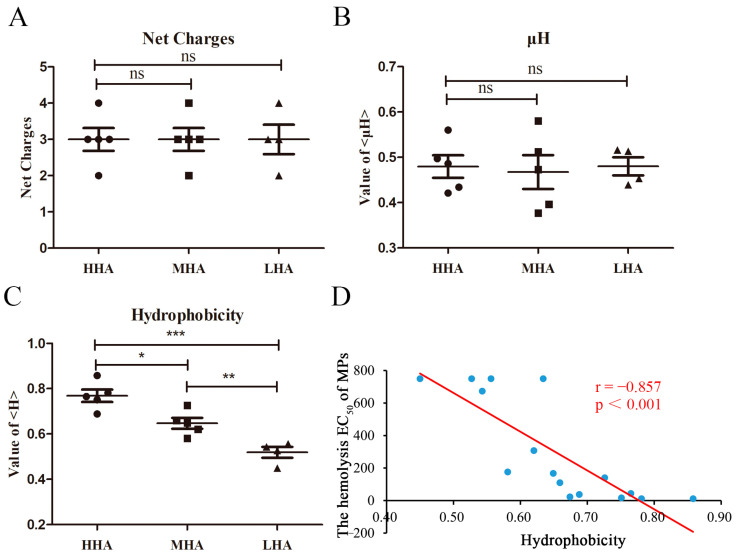
Structure–activity relationship analyses of the hemolytic effects of Protopolybia-MPIII and Protopolybia-MPI on HRBCs. The effect of net charges (**A**), amphipathicity (**B**) and hydrophobicity (**C**) of 14 mutations of Protopolybia-MPIII (5 HHA mutants, 5 MHA mutants and 4 LHA mutants) on hemolytic activity; ns: no significance, * indicates a *p* value < 0.05, ** indicates a *p* value < 0.01, and *** indicates a *p* value < 0.001. (**D**) The relationship between the hemolytic EC_50_ and hydrophobicity of 14 mutated peptides.

In previous studies, we divided 55 mastoparans into 4 subfamilies based on amino acid sequence alignments [15]. Therefore, we investigated the hemolytic activity of mastoparans in each subfamily and explored the relationship between the hemolytic activity and sequence homology of mastoparans (Figure 5A–D). We found that the characteristics of the hemolytic activity of mastoparans in the four subfamilies are quite diverse. The hemolytic activity of some mastoparans with close sequence homology is quite different, such as Dominulin-B and PMM2 with an identity of 86% (Figure 5A), Mastoparan-AF and Mastoparan-M with an identity of 92% (Figure 5B), and Mastoparan-X(V) and Mastoparan-like peptide 12b with an identity of 100% (Figure 5B). We also found that the proportion of HHA mastoparans in subfamily 3 (64.3%, 9/14) was much higher than that in total mastoparans (32.7%, 18/55) and other subfamilies (27.3% in subfamily 1, 29.4% in subfamily 2 and 10% in subfamily 4) (Figure 5A–D). The different amino acids were mainly located at the 9th to 12th amino acid sequences. Next, to further explore the amino acids that play key roles in the hemolytic activity, we selected 2 mastoparan peptides from subfamily 3 and both derived from *Protopolybia exigua*, Protopolybia-MPIII and Protopolybia-MPI, which have significant differences in hemolytic activity with only four different amino acid residue sites: sites 9, 11, 12 and 13 (Table 2). A total of 14 mutants were obtained based on the different amino acids (Table 2). The results showed that hydrophobicity, not net charge or amphipathicity, plays a critical role in the hemolytic activity of 14 Protopolybia-MPIII mutants (Figure 6A–D), and the hemolytic activity increased after the mutation of Protopolybia-MPIII 12D to 12A, 13A to 13I and 12D-13A to 12A-13I (Protopolybia-MPIII-3, Protopolybia-MPIII-4 and Protopolybia-MPIII-10), accompanied by an increase in the hydrophobicity of the mutants. The hemolytic activity of the 9 mutants significantly decreased, as shown in Table 2. The A9K and I11S mutations (Protopolybia-MPIII-1 and Protopolybia-MPIII-2) significantly decreased the hemolytic activity, and the A9K-I11S mutation (Protopolybia-MPIII-5, -13 and -14) almost completely eliminated the hemolytic activity, which corresponded to the significant decrease in hydrophobicity shown in Table 2. Structure–functional relationship studies of Protopolybia-MPIII and Protopolybia-MPI further showed that hydrophobicity significantly influences the hemolytic activity of the designed mastoparan peptides, which was consistent with the finding that hydrophobicity is a critical factor affecting the hemolytic activity of natural wasp mastoparan family peptides.

### 2.4. Combining the Reported Antimicrobial Activity with the Present Hemolytic Activity Data Highlights Four Peptides from the Wasp Mastoparan Family with Potential Antimicrobial Applications

Wasp mastoparan family peptides have promise for development into clinical drugs because of a wide variety of biological effects. Therefore, combining the antimicrobial activity of 34 mastoparans that have been reported and the hemolytic activity in our results, we found 4 mastoparans: Parapolybia-MP, Mastoparan-like peptide 12b, Dominulin A and Dominulin B (bold labelled) that have great clinical application prospects and high antibacterial activity (MIC ≤ 10 μM both on *Escherichia coli* and *Staphylococcus aureus/Bacillus subtilis*) and low hemolytic activity (EC_50_ > 400 μM). The selectivity index is one of the most suitable indicators for drug safety [17], the SI value of these 4 mastoparans all exceeded 40. We also found 5 mastoparans with great modification values in Table 3: Mastoparan-X(V), Mastoparan-C, Protonectarina-MP, Polybia-MPI and Polybia-MPII (red and underline labelled) with EC_50_ ≤400 μM and MIC < 10 μM, which should reduce hemolytic activity by lowering their hydrophobicity.

## 3. Discussion

Wasp stings are a serious problem worldwide, and patients in severe cases may experience multiorgan failure and even death. There are no specific detoxification drugs in the clinic. The clinical symptoms of wasp stings are mainly caused by allergic reactions and the direct toxicity of wasp venoms. The severity is closely related to the number of wasp stings, sting sites and wasp species. At present, comparative studies on venom toxicity among wasp species have not been reported. Si Hyeock Lee analysed the differences in proteins and peptides of social and solitary wasp venom [31], but the contents of various components in the venom are still unknown. The distribution of wasps also has geographical characteristics. For example, there are more than 6000 species of wasps in the world, and approximately 200 species have been found in China. Therefore, it is of great significance to explore the toxic characteristics of different wasp venoms for the regional control of wasps and clinical treatment of wasp stings.

Multiple studies have found that hemolysis plays a critical role in MODS induced by wasp stings. Mastoparan family peptides are one of the most important factors that induce hemolysis in wasp stings, and it is of great significance to elucidate the hemolytic characteristics of whole wasp mastoparan family peptides for the clinical treatment of wasp stings. Here, based on the library of 55 wasp mastoparan family peptides in a previous study, we systemically evaluated the hemolytic activity of each mastoparan on HRBCs and RRBCs. Our results showed that the hemolytic activity varies greatly among the 55 mastoparans; only 18 mastoparans had strong hemolytic activities on HRBCs (EC_50_ < 100 μM), and 23 mastoparans had slight hemolytic activities on HRBCs (EC_50_ > 400 μM). Our results showed that the proportion of HHA mastoparans from solitary wasp venom is much lower than that from social wasp venom. Our previous study found that the degranulation activity of mast cell induced by mastoparans from social wasp venom was higher than that from solitary wasp, which implies mastoparans from social wasp is more likely to trigger allergic reactions. Actually, the primary functions of venom from solitary and social wasps are different, solitary wasps sting their prey to paralyze and preserve it without killing, and social wasps usually sting to defend their colonies from predators [31]. The difference in hemolytic activity of mastoparans from a solitary wasp and a social wasp may be partially attributed to the difference in lifestyles and primary functions of wasp venom. At present, no comparative analysis of venom toxicity and the difference in venom compositions between wasp families has been reported. We found that the hemolytic activity in different wasp families is quite different. The proportion of HHA mastoparans in *Polistidae* was much higher than that in *Vespidae* (55.6% vs. 31.8%), and there were 9 HHA mastoparans derived from 5 *Polistidae* wasp venoms in our peptide library. High hemolytic activity was found in 80% (4/5) of *Polistidae* wasp venoms and 28.6% (4/14) of *Vespidae* wasp venoms, which indicated that the incidence of hemolysis induced by *Polistidae* wasp stings may be higher than that induced by *Vespidae* wasp stings (mastoparan family peptides from *Polistidae* may play a more important role in inducing hemolysis than those from *Vespidae*). However, the nests of *Vespidae* are generally larger and more complex than those of *Polistidae*, which means that *Vespidae* wasps live in a larger group and that humans are likely to be attacked by a larger number of wasps and receive more wasp stings, especially from *Agelaia pallipes pallipes*. In our results, Mastoparan-T2 (18.5 ± 4.8 μM), Mastoparan-T1 (49.3 ± 2.6 μM) and Mastoparan-T4 (45.0 ± 3.7 μM) are three of the top 10 mastoparans with high hemolytic activity, which are derived from *Vespa tropica*. Agelaia-MPI and Agelaia-MPII are two HHA mastoparans in *Agelaia pallipes pallipes* venom, and Agelaia-MPI (5.8 ± 0.2 μM) is the most active mastoparan with hemolytic activity in our results. Therefore, our data indicated that the venoms of *Polistidae* wasps, *Vespa tropica* and *Agelaia pallipes pallipes* are more likely to induce hemolysis, and additional attention should be given to the hemolysis of patients who have been attacked by these wasps.

There are three main mechanisms of mastoparan family peptide-induced hemolysis: insertion of a phospholipid bilayer and direct destruction of the cell membrane; activation of phospholipase A [7] and synergistic PLAs to induce cell membrane damage; and targeted inhibition of intracellular biochemical reactions and indirect induction of hemolysis [8]. In this study, we mainly studied the direct toxic effect of mastoparan family peptides on red blood cells and found great differences in hemolytic activity among 55 mastoparans. Hemolytic activity was positively correlated with the hydrophobicity of mastoparans, which plays a critical role in the interaction between mastoparan family peptides and the phospholipid bilayer. The effect on the activation of PLA and other biological functions associated with the hemolysis of cells needs further investigation.

Amidation at the C-terminal is an important feature of mastoparan family peptides. There are only four mastoparans without C-terminal amidation in our mastoparans library. Alessandra V.R. found that the C-terminal amidation of Protonectarina-MP promotes the stabilization and increases the contents of helical structure, which could enhance the interaction with phospholipid of animal and bacterial cell membranes, which means that the amidation may promote the antibacterial activity and hemolytic activity. This is consistent with present studies. All these data show that C-terminal amidation is critical for the biological effect of mastoparans and the mechanism needs further investigation.

Mastoparan family peptides have a wide variety of biological functions, including mast cell degranulation, antimicrobial and anticancer activities, and antimicrobial activity was the most widely studied in the past decade [14,15,22,32]. The unique cationic α-helix structure of wasp mastoparan family peptides, one of the most important motifs of antimicrobial peptides, suggests that mastoparan family peptides have a wide range of antimicrobial activities. A number of studies have reported that a series of mastoparan family peptides from different wasp venoms have significant antibacterial activity against both Gram-positive and Gram-negative bacteria, which suggests that mastoparan family peptides may be developed into new antibacterial agents in the future. However, hemolytic activity is an important factor limiting the development of mastoparan family peptides as new antimicrobial agents. Actually, the mechanisms of hemolysis and antibacterial activity induced by mastoparan family peptides were similar, and both could cause rupture of the cell membrane of red blood cells or bacteria. Here, we found that hydrophobicity plays a critical role in the hemolytic activity but not net charges or amphipathicity, both of which play a pivotal role in the antimicrobial activity of AMPs, and modifying the hydrophobicity of mastoparan family peptides may be an important way to reduce the side effects of mastoparan family peptides. Therefore, we analysed the antibacterial activity and hemolytic activity of 34 mastoparans in our library and found 4 mastoparans with high antibacterial activity and low hemolytic activity, which have great modification value.

## 4. Conclusions

In conclusion, our research systematically and comprehensively studied the hemolytic activity of 55 mastoparan family peptides and characterized four new leads for the antimicrobial application of mastoparans. Based on detailed structure–activity analyses, we found that hydrophobicity is a critical factor affecting the hemolytic activity of wasp mastoparan family peptides, which might provide a large chemical space to support the molecular design and molecular optimization of mastoparan family peptides with a net charge- and amphiphilicity-independent low hemolytic activity strategy in the future.

## 5. Materials and Methods

### 5.1. Peptide Synthesis and Bioinformatic Analysis

All mastoparan family peptides used in this study were synthesized and purified by ChinaPeptides Corporation (Shanghai, China) as previously described, and peptide purity all exceeded 95% and was determined by reverse-phase high-performance liquid chromatography (RP-HPLC). Fourteen mutants of Protopolybia-MPIII were designed based on the different amino acids between Protopolybia-MPIII and Protopolybia-MPI at sites 9, 11, 12 and 13. Peptides were stored as lyophilized powders before use. The physical characteristics of peptides, including net charges, hydrophobicity and amphipathicity were calculated online using the HeliQuest server [29,33]. DNAMAN software was used to obtain multiple amino acid sequence alignments of wasp mastoparans and homology trees [34].

### 5.2. Hemolytic Assay

Fresh human red blood cells were obtained from healthy donors and collected in a sterile blood collecting vessel covered with sodium citrate (1:9) as the anticoagulating agent. Rat red blood cells were collected from the abdominal aorta in sterile blood-collecting vessels coated with sodium citrate (1:4) after the SD rats were anaesthetized with isoflurane. Then, the cells were washed three times with phosphate-buffered saline via centrifugation (1000× *g*, 4 °C, 5 min) and prepared in a sterile 96-well polypropylene plate to achieve a final concentration of 5% (*v*/*v*). Twofold dilutions of the peptides were prepared in PBS to 100 μL and mixed with an equal volume of HRBCs/RRBCs suspension to final peptide concentrations ranging from 10 to 320 μg/mL. After coincubation at 37 °C for 1 h, the plate was centrifuged (2000× *g*, 4 °C, 20 min), and 100 μL of supernatant was taken for optical density measurements at 540 nm using a microtiter plate reader (SpectraMax 190). The HRBCs/RRBCs were assayed with PBS (blank) or 1% Triton X-100 (positive control) to represent 0% and 100% hemolysis, respectively. The percent hemolysis was calculated according to the following equation: Hemolysis Rate (%) = 100% × (A_mastoparans_ − A_blank_)/(A_Triton X-100_ − A_blank_). Three independent experiments were performed, and the mean hemolysis rate at each dose was used to calculate the EC_50_ in Sigmaplot software. The 50% effective concentration (EC_50_) and hemolysis rate at 320 μg/mL (the highest dose of mastoparans we used) were used as two indices to evaluate the hemolytic activity of mastoparans in HRBCs and RRBCs.

### 5.3. Statistical Analysis

Statistical analysis was performed with GraphPad Prism software (version 5.0, San Diego, CA, USA). A two-tailed Student’s t-test was used to calculate the statistical probability in this study. Differences in the data were considered to be statistically significant when the *p* value was equal to or less than 0.05. The curve of hemolysis induced by wasp mastoparans was generated in SigmaPlot 12.5 software, and the EC_50_ was calculated according to the equation (Four Parameter Logistic Curve): y = min + (max − min)/(1 + (x/EC_50_)^−Hillslope^). The heat map of hemolytic activity of mastoparan peptide was analysed in R software which is based on the value of log_2_(EC_50_). Then, the EC_50_ of LHA mastoparans on HRBC and RRBC was too low to calculate, and EC_50_ = 400 μM was used to conduct the heat map, which is the maximum EC_50_ of MHA and HHA mastoparan.

## Figures and Tables

**Table 1:** The hemolytic activity of 55 mastoparans from 31 wasp species on HRBCs and RRBCs.

Family	Species	Peptides	Sequences	<H> ^c^	<μH> ^d^	Z ^b^	HRBC			RRBC		
							Hemolysis ^a^	EC_50_ (μM)	R^2^	Hemolysis ^a^	EC_50_ (μM)	R^2^
*Eumenidae*	*Anterhynchium flavormarginatum micado*	EMP-AF	INLLKIAKGIIKSL-NH_2_	0.643	0.559	4	100%	110.6 ± 9.8	0.9803	100%	122.1 ± 13.1	0.9872
	*Eumenes fraterculus*	EMP-EF	FDVMGIIKKIASAL-NH_2_	0.655	0.489	2	100%	125.2 ± 20.6	0.9607	48%	216.8 ± 1.6	0.9997
	*Eumenes rubrofemoratus*	EMP-ER	FDIMGLIKKVAGAL-NH_2_	0.651	0.493	2	25%			46%	230.6 ± 3.7	0.9997
	*Eumenes* *rubronotatus*	Eumenitin-R	LNLKGLIKKVASLLN	0.508	0.498	4	5%			7%		
		Eumenitin-F	LNLKGLFKKVASLLT	0.565	0.461	4	67%	157.1 ± 2.6	0.9995	44%	207.1 ± 2.0	0.9998
		Eumenitin	LNLKGIFKKVASLLT	0.571	0.465	4	6%			4%		
	*Eumenes * *pomiformis*	EpVP1	INLKGLIKKVASLLT	0.572	0.455	4	9%			0%		
		EpVP2a	FDLLGLVKKVASAL-NH_2_	0.633	0.457	2	79%	151.9 ± 6.3	0.9972	41%	238.8 ± 7.6	0.9985
		EpVP2b	FDLLGLVKSVVSAL-NH_2_	0.766	0.439	1	100%	34.1 ± 3.5	0.9908	100%	50.6 ± 2.6	0.9951
	*Eumenes micado*	EMP-EM1	LKLMGIVKKVLGAL-NH_2_	0.686	0.52	4	21%			7%		
		EMP-EM2	LKLLGIVKKVLGAI-NH_2_	0.727	0.54	4	8%			2%		
	*Orancistrocerus* *drewseni*	EMP-OD	GRILSFIKGLAEHL-NH_2_	0.589	0.632	2	2%			49%	189.8 ± 32.0	0.9777
		OdVP3a	KDLHTVVSAILQAL-NH_2_	0.595	0.562	1	24%			27%		
*Vespidae*	*Vespula lewisii*	Mastoparan (L)	INLKALAALAKKIL-NH_2_	0.576	0.398	4	100%	82.9 ± 3.8	0.9969	41%	242.5 ± 2.6	0.9999
	*Vespula vulgaris*	Mastoparan-V1	INWKKIKSIIKAAMN-NH_2_	0.407	0.428	5	9%			16%		
		Mastoparan-V2	INWKKIKSLIKAAMS-NH_2_	0.437	0.388	5	15%			52%	182.4 ± 3.6	0.9994
	*Vespa bicolor*	MP-VB1	INMKASAAVAKKLL-NH_2_	0.377	0.234	4	0%			7%		
		MP-VB2	INMKAVAAVAKKPL-NH_2_	0.397	0.261	4	0%			0%		
	*Vespa. xanthoptera*	Mastoparan-X(V)	INWKGIAAMAKKLL-NH_2_	0.56	0.419	4	29%	349.4 ± 4.9	0.9999	70%	156.5 ± 2.4	0.9996
	*Vespa analis*	Mastoparan-A	IKWKAILDAVKKVL-NH_2_	0.541	0.538	4	7%			56%	183.0 ± 3.4	0.9994
	*Vespa basalis*	Mastoparan-B	LKLKSIVSWAKKVL-NH_2_	0.461	0.404	5	22%			19%		
	*Vespa crabro*	Mastoparan-C	INLKALLAVAKKIL-NH_2_	0.641	0.392	5	100%	30.2 ± 1.3	0.9972	100%	64.4 ± 10.7	0.9984
	*Vespa orientalis*	Mastoparan-II	INLKALAALVKKVL-NH_2_	0.6	0.416	4	78%	134.6 ± 1.2	0.9999	90%	146.8 ± 3.4	0.9993
		HR1	INLKAIAALVKKVL-NH_2_	0.607	0.423	4	56%	197.7 ± 8.9	0.9976	39%	253.4 ± 13.0	0.9983
	*Vespa tropica*	Mastoparan-T(D)	INLKAIAAFAKKLL-NH_2_	0.583	0.402	4	15%	-	-	19%	-	-
		Mastoparan-T1	INLKVFAALVKKFL-NH_2_	0.712	0.442	4	100%	30.8 ± 1.6	0.9964	87%	22.7 ± 7.1	0.9735
		Mastoparan-T2	INLKVFAALVKKLL-NH_2_	0.706	0.436	4	100%	11.8 ± 3.1	0.9727	100%	21.1 ± 2.4	0.9886
		Mastoparan-T3	INLRGFAALVKKFL-NH_2_	0.624	0.466	4	100%	112.1 ± 8.0	0.9911	100%	51.6 ± 2.1	0.9987
		Mastoparan-T4	INLFGFAALVKKFL-NH_2_	0.824	0.31	3	100%	28.5 ± 2.3	0.9924	100%	17.6 ± 2.4	0.9765
	*Vespa magnifica*	Mastoparan-like peptide 12b	INWKGIAAMKKLL-NH_2_	0.579	0.348	4	0%	-	-	0%	-	-
	*Vespa mandarinia*	Mastoparan-M	INLKAIAALAKKLL-NH_2_	0.576	0.399	4	18%	-	-	28%	-	-
	*Vespa affinis*	Mastoparan AF	INLKAIAALAKKLF-NH_2_	0.583	0.4	4	47%	227.0 ± 3.1	0.9999	92%	107.3 ± 15.7	0.9834
	*Agelaia pallipes * *pallipes*	Agelaia-MPI	INWLKLGKAIIDAL-NH_2_	0.716	0.538	2	100%	3.7 ± 0.14	0.9977	98%	7.0 ± 0.7	0.9912
		Agelaia-MPII	INWKAILQRIKKML-NH_2_	0.556	0.568	5	100%	44.8 ± 10.4	0.9767	97%	62.1 ± 3.8	0.997
	*Mischocyttarus phthisicus*	MP	INWLKLGKKMMSAL-NH_2_	0.594	0.51	4	87%	123.6 ± 15.3	0.9815	10%	-	-
*Polybiidae*	*Protopolybia* *exigua*	Protopolybia-MP I	INWLKLGKKVSAIL-NH_2_	0.634	0.439	5	5%	-	-	5%	-	-
		Protopolybia-MPII	INWKAIIEAAKQAL-NH_2_	0.511	0.389	2	13%	-	-	24%	-	-
		Protopolybia-MPIII	INWLKLGKAVIDAL-NH_2_	0.674	0.506	2	96%	61.9 ± 8.3	0.9857	93%	34.6 ± 2.8	0.9981
	*Protonectarina sylveirae*	Protonectarina-MP	INWKALLDAAKKVL-NH_2_	0.497	0.474	3	100%	85.2 ± 5.9	0.9928	35%	326.5 ± 26.4	0.9995
	*Polybia paulista*	Polybia-MP I	IDWKKLLDAAKQIL-NH_2_	0.489	0.511	2	56%	176.6 ± 7.0	0.9982	100%	51.4 ± 2.2	0.9961
		Polybia-MP II	INWLKLGKMVIDAL-NH_2_	0.74	0.457	2	91%	23.3 ± 1.4	0.9961	100%	29.1 ± 1.5	0.9957
		Polybia-MP III	IDWLKLGKMVMDVL-NH_2_	0.752	0.448	1	79%	38.5 ± 5.9	0.9854	100%	74.8 ± 10.4	0.979
		Polybia-MP IV	IDWLKLRVISVIDL-NH_2_	0.829	0.222	1	0%	-	-	0%	-	-
		Polybia-MP V	INWHDIAIKNIDAL-NH_2_	0.584	0.42	0	0%	-	-	0%	-	-
	*Parapolybia indica*	Parapolybia-MP	INWKKMAATALKMI-NH_2_	0.545	0.412	4	32%	306.4 ± 162.6	0.9627	22%	-	-
*Ropalidiidae*	*Ropalidia*	Ropalidia-MP	INWAKLGKLALQAL-NH_2_	0.641	0.34	3	100%	42.5 ± 1.7	0.9978	94%	122.2 ± 4.3	0.9979
*Polistidae*	*Polistes jadwigae*	Mastoparan-J	VDWKKIGQHILSVL-NH_2_	0.629	0.519	2	100%	69.5 ± 3.2	0.9979	100%	60.1 ± 1.3	0.9994
	*Polistes major major*	PMM2	INWKKIASIGKEVLKAL-NH_2_	0.45	0.499	4	100%	42.6 ± 2.5	0.9933	100%	80.9 ± 6.4	0.9909
	*Polistes dorsalis*	PDD-A	INWKKIFQKVKNLV-NH_2_	0.457	0.565	5	30%	353.5 ± 44.8	0.9992	27%	-	-
		PDD-B	INWLKLGKKILGAL-NH_2_	0.671	0.555	5	100%	48.5 ± 3.4	0.9945	100%	55.6 ± 4.1	0.9913
	*Polistes dominulus*	Dominulin A	INWKKIAEVGGKILSSL-NH_2_	0.488	0.493	3	4%	-	-	0%	-	-
		Dominulin B	INWKKIAEIGKQVLSAL-NH_2_	0.495	0.462	3	8%	-	-	0%	-	-
	*Polistes rothneyi * *iwatai*	Pm-R1	INWLKLGKKILGAI-NH_2_	0.678	0.563	4	100%	72.0 ± 8.5	0.9859	92%	105.1 ± 2.2	0.9991
		Pm-R2	LNFKALAALAKKIL-NH_2_	0.576	0.408	4	66%	165.9 ± 8.0	0.9967	34%	249.5 ± 9.9	0.9986
		Pm-R3	INWLKLGKQILGAL-NH_2_	0.726	0.511	3	100%	32.3 ± 1.7	0.9957	100%	73.5 ± 5.1	0.9939

(a) Hemolysis rate of mastoparans at 320 μg/mL; (b) net charges were obtained from HiliQuest server; (c) <H>: mean hydrophobicity value represents the sum of all residue hydrophobicity indices divided by the number of residues; (d) <μH>: the hydrophobic moment of each peptide is relative to the hydrophobic moment of the peptide with perfect amphipathicity. The hemolytic activity was too low to calculate the EC_50_.

**Table 2:** The physical properties and hemolytic activity of Protopolybia-MPIII and mutants.

Peptides	Sequences	pI	<H>	<μH>	Z	HRBC		RRBC	
						EC_50_ (μM)	R^2^	EC_50_ (μM)	R^2^
Protopolybia-MPIII	INWLKLGKAVIDAL-NH_2_	8.59	0.674	0.506	2	23.0 ± 1.2	0.9968	42.8 ± 2.1	0.991
Protopolybia-MPI	INWLKLGKKVSAIL-NH_2_	10.30	0.634	0.439	4	-	-	-	-
Protopolybia-MPIII-1	INWLKLGKKVIDAL-NH_2_	9.70	0.581	0.580	3	176.4 ± 1.0	0.9999	158.4 ± 2.3	0.9996
Protopolybia-MPIII-2	INWLKLGKAVSDAL-NH_2_	8.59	0.543	0.453	2	673.3 ± 189.0	0.9999	639.5 ± 1.6	0.9998
Protopolybia-MPIII-3	INWLKLGKAVIAAL-NH_2_	10.00	0.751	0.434	3	16.8 ± 1.0	0.9959	21.5 ± 1.2	0.9963
Protopolybia-MPIII-4	INWLKLGKAVIDIL-NH_2_	8.59	0.781	0.497	2	11.3 ± 6.2	0.9021	19.8 ± 1.2	0.9495
Protopolybia-MPIII-5	INWLKLGKKVSDAL-NH_2_	9.70	0.450	0.513	3	-	-	-	-
Protopolybia-MPIII-6	INWLKLGKKVIAAL-NH_2_	10.30	0.659	0.512	4	110.0 ± 2.5	0.9992	107.6 ± 3.2	0.9927
Protopolybia-MPIII-7	INWLKLGKKVIDIL-NH_2_	9.70	0.688	0.560	3	37.8 ± 3.7	0.9908	57.5 ± 2.7	0.9967
Protopolybia-MPIII-8	INWLKLGKAVSAAL-NH_2_	10.00	0.620	0.377	3	307.8 ± 6.2	0.9999	444.0 ± 2.1	0.9999
Protopolybia-MPIII-9	INWLKLGKAVSDIL-NH_2_	8.59	0.649	0.473	2	167.3 ± 2.7	0.9994	161.7 ± 1.3	0.9996
Protopolybia-MPIII-10	INWLKLGKAVIAIL-NH_2_	10.00	0.858	0.421	3	11.3 ± 1.2	0.9899	11.7 ± 1.0	0.9883
Protopolybia-MPIII-11	INWLKLGKAVSAIL-NH_2_	10.00	0.726	0.396	3	140.6 ± 2.4	0.9996	133.6 ± 2.2	0.9993
Protopolybia-MPIII-12	INWLKLGKKVIAIL-NH_2_	10.30	0.765	0.486	4	43.9 ± 3.6	0.9928	55.2 ± 2.2	0.9978
Protopolybia-MPIII-13	INWLKLGKKVSDIL-NH_2_	9.70	0.556	0.516	3	-	-	-	-
Protopolybia-MPIII-14	INWLKLGKKVSAAL-NH_2_	10.30	0.527	0.439	4	-	-	-	-

-: the hemolytic activity was too low to calculate the EC_50_.

**Table 3:** The statistics of antibacterial activity and hemolytic activity of 34 mastoparans.

Mastoparan	MIC (μM)			EC_50_ (μM)		SI		Refs.
	*E. coli*	*S. aureus*	*B. subtilis*	HRBC	RRBC	*E. coli*	*S. aureus*	
EMP-AF	13	3.3	ND	110.6 ± 9.8	122.1 ± 13.1	8.5	33.5	[18]
EMP-EF	30	30	ND	125.2 ± 20.6	216.7 ± 1.6	4.2	4.2	[13]
Eumenitin-R	30	60	ND	-	-	>13.3	6.7	[13]
EMP-ER	30	30	ND	-	230.6 ± 3.7	>13.3	13.3	[13]
Eumenitin-F	30	>60	ND	157.1 ± 2.6	207.1 ± 2.0	5.2	/	[13]
Eumenitin	6	6	>60	-	-	>66.7	>66.7	[19]
EMP-OD (OdVP1)	97	97	10	-	189.8 ± 32.0	>4.1	>4.1	[20]
OdVP3	>200	>200	10	-	-	/	/	[20]
EpVP1	25	100	5	-	-	>16.0	>4.0	[20]
EpVP2a	100	50	5	151.9 ± 6.3	238.8 ± 7.6	1.5	3.0	[20]
EpVP2b	200	25	5	34.1 ± 3.5	50.6 ± 2.6	0.2	1.4	[20]
Mastoparan-L	50	5	8	82.9 ± 3.8	242.5 ± 2.6	1.7	16.6	[21]
MP-VB1	10.3	2.5	ND	-	-	>38.8	>160.0	[22]
Mastoparan-X(V)	4.8	2.4	ND	349.4 ± 4.9	312.1 ± 4.7	72.8	145.6	[23]
Mastoparan-A	5	20	ND	-	183.0 ± 3.4	>80.0	>20.0	[23]
Mastoparan-B	10	60	ND	-	-	>40.0	>6.7	[23]
Mastoparan-C	4	4	ND	30.2 ± 1.3	64.4 ± 10.7	7.6	7.6	[22]
Mastoparan-T(D)	5.3	15.9	ND	-	-	>75.5	>25.2	[23]
Protonectarina-MP	5	2.5	2.5	85.2 ± 5.9	326.5 ± 26.4	17.0	34.1	[24]
Polybia-MP I	4.8	9	2.4	176.6 ± 7.0	51.4 ± 2.2	36.8	19.6	[25]
Polybia-MP II	5	2.5	5	23.3 ± 1.4	29.1 ± 1.5	4.7	9.3	[26]
Polybia-MP III	ND	19	ND	38.5 ± 5.9	74.8 ± 10.4	/	2.0	[26]
**Parapolybia-MP**	**2.4**	**1.8**	**2.2**	**-**	**-**	>166.7	>222.2	[24]
Agelaia-MPI	243.8	121.8	151.2	3.7 ± 0.14	7.0 ± 0.7	0.02	0.04	[12]
**Mastoparan-like peptide 12b**	**10**	**2.5**	**ND**	**-**	**-**	>40.0	>160.0	[27]
Mastoparan-M	13.5	3.38	ND	-	-	>29.6	>118.3	[23]
Mastoparan-AF	2.64	10.6	ND	227.0 ± 3.1	107.3 ± 15.7	86.0	21.4	[23]
PDD-A	7.5	ND	11.8	353.5 ± 44.8	-	47.1	/	[28]
PDD-B	70	ND	15.5	48.5 ± 3.4	55.6 ± 4.1	0.7	/	[28]
MP	65	ND	9	123.6 ± 15.3	-	1.9	/	[28]
EMP-EM1	7	34	68	-	-	>57.1	>11.8	[29]
EMP-EM2	3	17	68	-	-	>133.3	>23.5	[29]
**Dominulin A**	**1**	**ND**	**4**	**-**	**-**	>400.0	/	[30]
**Dominulin B**	**1**	**ND**	**4**	**-**	**-**	>400.0	/	[30]

SI: selectivity index, SI = EC_50_ of hemolytic activity on HRBC/MIC*_E. coli_* or MIC *_S. aureus_*. ND means no data; -: the hemolytic activity was too low to calculate the EC_50_. /: the value of SI is unpredictable.

## Data Availability

Data will be available upon request from the author.

## Supplementary Materials

The following supporting information can be downloaded at: https://www.mdpi.com/article/10.3390/toxins15100591/s1, Figure S1: Characterization of the hemolytic activity of representative wasp mastoparan peptides with high activity and modest activity on human blood red cells; Figure S2: Characterization of the hemolytic activity of representative wasp mastoparan peptides with high activity and modest activity on Rat blood red cells.
