# Supplementary material for: Characterization of the Hemolytic Activity of Mastoparan Family Peptides from Wasp Venoms
Source: Toxins (Basel). 2023 Sep 28;15(10):591. doi: 10.3390/toxins15100591 (PMC10611374; doi:10.3390/toxins15100591)
Supplement: Supplementary file 1 [file toxins-15-00591-s001.zip › toxins-2581965-supplementary.pdf]

Supplementary Materials: Characterization of the Hemolytic Activity of Mastoparan Family Peptides from Wasp Venoms

Xiangdong Ye, Huajun Zhang, Xudong Luo, Fengyin Huang, Fang Sun, Liangbin Zhou, Chenhu Qin, Li Ding, Haimei Zhou, Xin Liu, Zongyun Chen

Figure S1.

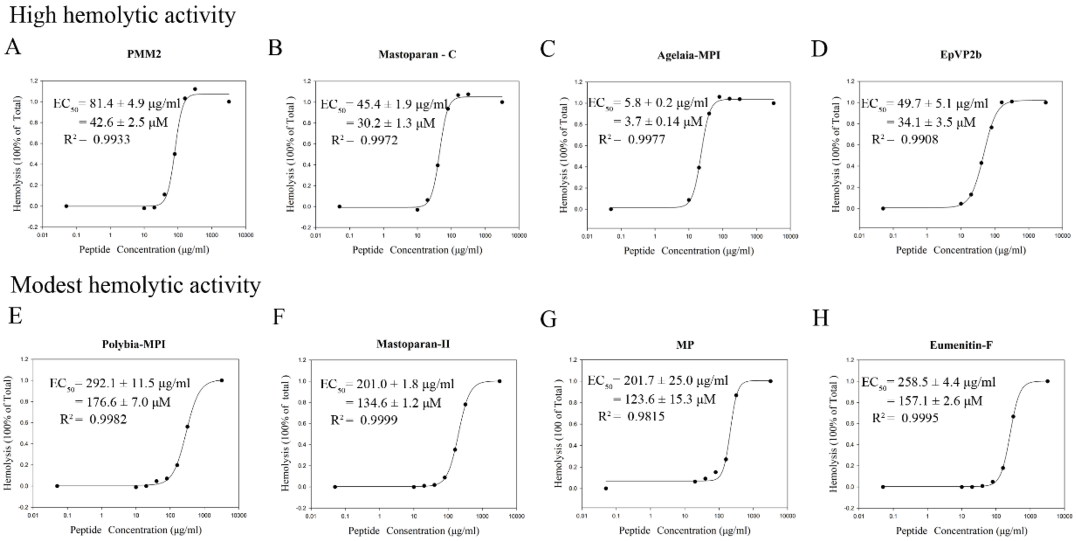

Figure S1. Characterization of the hemolytic activity of representative HHA and MHA mastoparan peptides on human blood red cells. (A–H) Hemolysis curve of HRBCs induced by four HHA mastoparans: PMM2 (A), Mastoparan-C (B), Agelaia-MPI (C), EpVP2b (D), and four MHA mastoparans: Polybia-MPI (E), Mastoparan-II (F), MP (G) and Eumenitin-F (H). Curves were generated by SigmaPlot 12.5 software, and EC_{50} values were calculated according to the equation (four-parameter logistic curve): $y = min + (max - min)/ (1 + (x/EC_{50})^{-Hillslope})$.

Figure S2.

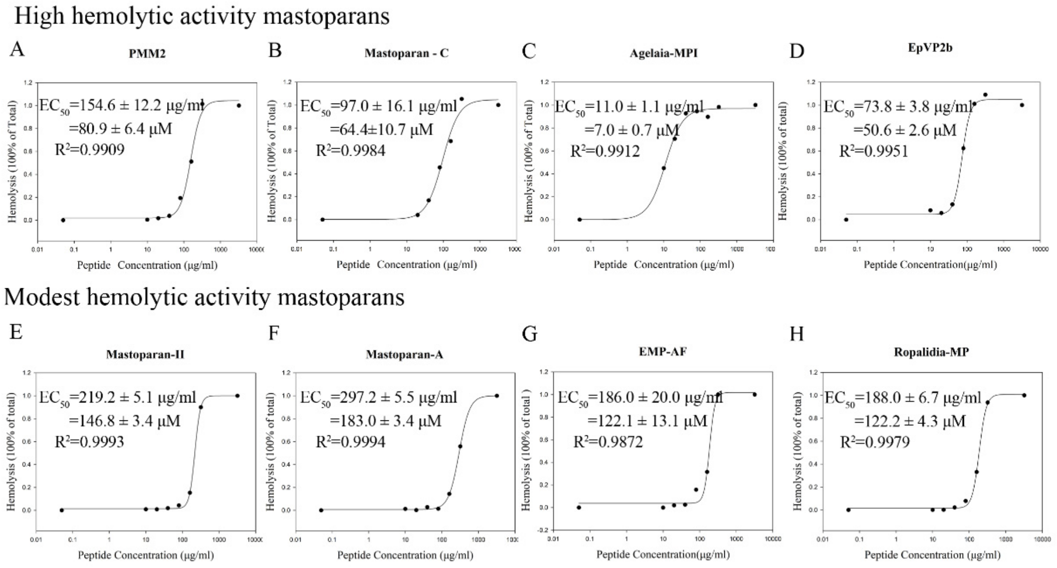

Figure S2. Characterization of the hemolytic activity of representative wasp mastoparan peptides with high activity and modest activity on Rat blood red cells. (A–H) Hemolysis curve of RRBCs induced by four HHA mastoparans: PMM2 (A), Mastoparan-C (B), Agelaia-MPI (C), EpVP2b (D), and four MHA mastoparans: Mastoparan-II (E), Mastoparan-A (F), EMP-AF (G) and Ropalidia-MP (H). Curves were generated in SigmaPlot 12.5 software, and EC_{50} values were calculated according to the following equation (four-parameter logistic curve): $y = min + (max - min)/(1 + (x/EC_{50}) - Hillslope)$.
